# Extra-hepatic comorbidity burden significantly increases 90-day mortality in patients with cirrhosis and high model for endstage liver disease

**DOI:** 10.1186/s12876-020-01448-z

**Published:** 2020-09-16

**Authors:** Scott Coppel, Karan Mathur, Burcin Ekser, Kavish R. Patidar, Eric Orman, Archita P. Desai, Eduardo Vilar-Gomez, Chandrashekhar Kubal, Naga Chalasani, Lauren Nephew, Marwan Ghabril

**Affiliations:** 1 Medicine, Indiana University, Indianapolis, IN USA; 2 Gastroenterology and Hepatology, Indiana University, 702 Rotary Circle, suite 225, Indianapolis, IN 46202 USA; 3 Transplant Surgery, Indiana University, Indianapolis, IN USA

**Keywords:** Cirrhosis, Comorbidity, Charlson comorbidity index, Liver transplantation, Mortality

## Abstract

**Background:**

We examined how extra-hepatic comorbidity burden impacts mortality in patients with cirrhosis referred for liver transplantation (LT).

**Methods:**

Adults with cirrhosis evaluated for their first LT in 2012 were followed through their clinical course with last follow up in 2019. Extra-hepatic comorbidity burden was measured using the Charlson Comorbidity Index (CCI). The endpoints were 90-day transplant free survival (Cox-Proportional Hazard regression), and overall mortality (competing risk analysis).

**Results:**

The study included 340 patients, mean age 56 ± 11, 63% male and MELD-Na 17.2 ± 6.6. The CCI was 0 (no comorbidities) in 44%, 1–2 in 44% and > 2 (highest decile) in 12%, with no differences based on gender but higher CCI in patients with fatty and cryptogenic liver disease. Thirty-three (10%) of 332 patients not receiving LT within 90 days died. Beyond MELD-Na, the CCI was independently associated with 90-day mortality (hazard ratio (HR), 1.32 (95% confidence interval (CI) 1.02–1.72). Ninety-day mortality was specifically increased with higher CCI category and MELD ≥18 (12% (CCI = 0), 22% (CCI = 1–2) and 33% (CCI > 2), (*p* = 0.002)) but not MELD-Na ≤17. At last follow-up, 69 patients were alive, 100 underwent LT and 171 died without LT. CCI was associated with increased overall mortality in the competing risk analysis (Sub-HR 1.24, 95%CI 1.1–1.4).

**Conclusions:**

Extra-hepatic comorbidity burden significantly impacts short-term mortality in patients with cirrhosis and high MELD-Na. This has implications in determining urgency of LT and mortality models in cirrhosis and LT waitlisting, especially with an ageing population with increasing prevalence of fatty liver disease.

## Background

Cirrhosis is a serious consequence of chronic liver diseases, and represents a substantial burden of morbidity, mortality and health-care expenditure. It carries a poor prognosis in the setting of decompensation or development of hepatocellular carcinoma, with liver transplantation (LT) being the only definitive and lifesaving therapy. In this context, extra-hepatic comorbidities may carry multiple hazards to patients with cirrhosis in need of LT. They carry direct risk of mortality related to the impact of comorbidity [1, 2], as well as risk of precluding candidacy for lifesaving LT [3], and even risk of post LT mortality [4].

The Charlson Comorbidity Index (CCI) is a well described and validated instrument determined by the presence, and in some cases severity, of 16 comorbid conditions, including liver disease [5]. The CCI predicts 1-year mortality in general populations and in patients with organ specific disease such as acute and chronic heart disease [5–8]. Both liver disease severity, reflected in MELD, and HCC are known to impact mortality and LT considerations in patients with cirrhosis. Beyond MELD, CCI predicts mortality in patients with suspected drug-induced liver injury [9]. However, the impact of extra-hepatic comorbidity burden on *short-term* and overall mortality in patients with advanced cirrhosis referred for LT has not been well-studied or quantified. The assessment of overall comorbidity burden, rather than individual comorbid conditions considered by transplant centers, may provide an aggregate measure of risk posed by the burden of extra-hepatic conditions.

In this study we measured the extra-hepatic medical comorbidity burden in a cohort of consecutive patients referred for LT using CCI (excluding the contributions of liver disease and HCC). The aims of the study were to determine the impact of extra-hepatic comorbidity burden on 90-day mortality and overall mortality in the study cohort.

## Patients and methods

### Patients

This study was approved by the Indiana University institutional review board, and was performed and reported per standardized reporting guidelines for qualitative research [10]. All patients with cirrhosis evaluated for LT at our center in 2012 were assessed. Patients were followed from the time of initial assessment through their pre and post-LT course until last follow-up in 2019. The selection of the study period was designed to allow for a relatively long post-LT follow-up (anticipated 5 years or more).

Patients with prior LT, absence of cirrhosis, or referred for multi-organ transplant were excluded. Demographic and clinical data were collected, including age, gender, body mass index (BMI), race, and etiology of liver disease. The severity of liver disease was measured using the MELD sodium equation (MELD-Na) [11]. Patients were followed until last contact for survivors or until death.

### Comorbidity burden

Extra-hepatic comorbidity burden was measured using the CCI (contributions of liver disease and HCC to the malignancy component were excluded). For example, a patient with no extra-hepatic comorbidities but with cirrhosis and HCC would have a CCI of 0. The CCI was chosen as an easily calculated and widely recognized comorbidity score that is validated in multiple populations including patients with chronic and acute liver disease [5, 9, 12].

The CCI was analyzed primarily as a continuous variable in all risk-models. In addition, to examine the impact of low and high comorbidity burden we described outcomes within CCI categories using the following two thresholds; (i) patients with CCI = 0, a physiological reference group with the lowest extra-hepatic comorbidity burden, and (ii) the highest decile of CCI (> 2 in this cohort) to reflect the impact of the highest comorbidity burden. Patients with CCI = 1–2 represented an intermediate comorbidity burden group.

### Study endpoints

The cohort was followed through the LT evaluation process with long-term follow up. The primary outcomes were *90-day (short-term) mortality without LT* from the time of initial evaluation, which was examined in patients not undergoing LT within 90-days. The secondary outcome was o*verall mortality (longer-term) with LT as a competing risk,* which was examined in all patients.

### Additional analyses

As a means of sensitivity testing for the association of comorbidity burden with mortality, we repeated all analyses while measuring comorbidities with an alternate score to CCI. The extra-hepatic comorbidity burden was measured and analyzed using the Cirrhosis Comorbidity score (CIRCOM), which has been validated in patients with cirrhosis [2]. Both CCI and CIRCOM are validated in patients with liver disease but they measure comorbidity burden somewhat differently. While CCI is a simple additive score, CIRCOM is designed as a conditional model based on priority scores and variable inclusion of specific groups of conditions. The individual comorbid conditions and their relative weights in CCI and CIRCOM are also not uniformly shared nor equally weighted (Supplemental Table 1). We examined the associations of the two scoring systems and their component conditions with short and long-term mortality in this cohort.

Finally, we examined the impact of extrahepatic comorbidity burden on LT eligibility after excluding patients with obvious prohibitive conditions and those deemed too early for or declining evaluation for LT.

### Statistical methods

The analyses for factors associated with the study endpoints were performed using univariable and multivariable Cox proportional hazards regression for 90-day mortality, and univariable and multivariable competing risk regression for overall mortality, with LT as competing risk. The analyses were adjusted for age, gender, BMI, race, etiology of liver disease, MELD-Na and HCC. All analyses were two-sided with significance set at a *p*-value< 0.05, and were performed using SPSS 26 (IBM Corp. Released 2017. IBM SPSS Statistics for Windows, Version 26.0. Armonk, NY: IBM Corp) or Stata SE 16 (StataCorp. 2019. Stata Statistical Software: Release 16. College Station, TX: StataCorp LLC).

## Results

Among the 387 patients evaluated for LT 47 were excluded, including 30 patients who were referred for multi-organ transplant, 7 with prior LT and 10 without underlying cirrhosis. The remaining 340 patients met the inclusion criteria. Baseline demographic and clinic characteristics are summarized in Table 1. The mean CCI was 1 ± 1.2 and the median CCI was 1 (Interquartile range (IQR) 0, 1), with a score distribution of 0 in 44%, 1 to 2 in 44%, and > 2 in 12% of patients. There were no differences in mean CCI in females and males, although females had a trend for higher frequency of connective tissue disease (6.2% vs. 2.3%, (*p* = 0.06)). Mean CCI and patient age differed significantly according to etiology of cirrhosis (fatty liver (1.3 ± 1.1 and 61 ± 8), cryptogenic (0.9 ± 1.4 and 62 ± 8), viral (1.1 ± 1.3 and 57 ± 8), alcohol with viral (1.1 ± 1.4 and 54 ± 7), alcohol (0.6 ± 0.8 and 53 ± 10) and autoimmune (0.5 ± 1.1 and 51 ± 15) disease (*p* = 0.003 and < 0.001), respectively). While CCI did not correlate with patient age per se (Pearson coefficient 0.08, (*p* = 0.15)), mean age increased with higher CCI category (55 ± 10 with CCI = 0, 56 ± 9 with CCI = 1–2 and 59 ± 7 with CCI > 2) (*p* = 0.025).

**Table 1:** Baseline demographic and clinical characteristics of the 340 patients evaluated for liver transplantation for complications of cirrhosis. Data are shown as mean ± standard deviation or percentage unless otherwise specified. Patients who died within 90-days without LT and those who survived at least 90-days without LT are compared (8 patients underwent LT within 90-days and were excluded)

Demographic and clinical characteristics	Patients assessed ***N*** = 340	Patients who died within 90 days ***n*** = 33	Patients who survived 90-days without LT ***n*** = 297	***p***-value
Age	56 ± 11	57 ± 9	56 ± 9	0.5
Gender (male) (%)	63	21	40	0.04
Race (%)				
White	90	85	91	0.6
Black	5	9	4	
Hispanic	3	6	3	
Asian	1	None	1	
Other	1	None	1	
Body mass index	30 ± 7	30 ± 8	30 ± 6	0.7
Etiology of liver disease (%)				
Alcohol	19	12	20	0.4
Alcohol and viral	16	24	16	
Viral	32	39	31	
Autoimmune	8	None	8	
Fatty liver	21	21	21	
Cryptogenic	2	3	2	
MELD-Na	17.2 ± 6.6	25.1 ± 7.3	16.2 ± 5.8	< 0.001
Hepatocellular carcinoma (%)	24	30	23	0.4
CCI (mean)	1 ± 1.2	1.4 ± 1.2	1 ± 1.2	0.09
(median (IQR))	1 (0,1)	1 (0, 2)	1 (0,1)	
CCI category (%)				
CCI = 0	44	30	46	0.07
CCI = 1–2	44	52	43	
CCI > 2	12	18	110.	
CIRCOM (mean)	0.8 ± 1.2	1.6 ± 1.4	0. ± 1.1	0.001
(median (IQR))	0 (0,1)	1 (0, 3)	0 (0,1)	

*Abbreviations*: *CCI* Charlson Comorbidity Index (excluding liver disease and liver cancer), *IQR* interquartile range, *LT* liver transplantation, *MELD-Na* Model for End-stage Liver Disease with sodium modification

### The impact of comorbidity burden on 90-day survival

Among the 340 patients evaluated 33 died within 90 days without LT, while 8 underwent LT within 90-days and were excluded from this specific analysis. Causes of death included multiorgan failure (7), infection (4), gastrointestinal bleed (4), cardiac (3), stroke (1) and undetermined (14). The CCI was independently associated with increased 90-day mortality on multivariable Cox regression analysis (Table 2), as was MELD-Na. The factors not associated with 90-day mortality included age, gender, BMI, race, etiology of liver disease and HCC. The results were similar when excluding 7 patients with moderate to severe renal disease (contributing to CCI) which also contributed to higher MELD-Na. Extra-hepatic comorbidity burden as measured by CIRCOM was associated with an almost identical 90-day mortality risk (adjusted HR 1.34, 95%CI 1.02–1.77).

**Table 2:** The analysis of factors associated with 90-day mortality without liver transplant (LT) by Cox regression in 332 patients with cirrhosis referred for LT, 33 of whom died within 90 days of initial evaluation. Eight of 340 patients assessed underwent LT within 90-days from initial assessment and were excluded from this analysis

Factor	Univariable analysis			Multivariable analysis		
	Hazard ratio	95% confidence interval	***p***-value	Hazard ratio	95% confidence interval	***P***-value
CCI	1.2	(0.97–1.5)	0.09	1.33	(1.02–1.73)	0.037
MELD-Na	1.22	(1.16–1.29)	< 0.001	1.26	(1.18–1.33)	< 0.001
Age	1.01	(0.98–1.06)	0.5	1.02	(0.96–1.07)	0.5
Male gender	2.4	(1.03–5.4)	0.043	1.2	(0.5–3)	0.6
Body mass index	1.01	(0.96–1.07)	0.4	1.02	(0.96–1.09)	0.4
Race (reference white)						
Black	2.2	(0.7–7.1)	0.2	2.9	(0.8–11)	0.12
Hispanic	1.9	(0.5–7.9)	0.4	0.4	(0.1–1.7)	0.19
Asian	NA^a^			NA^a^		
Etiology liver disease (Reference alcohol)						
Alcohol and viral	2.4	(0.7–8)	0.15	2.8	(0.8–9.8)	0.08
Viral	1.9	(0.6–6)	0.24	2.3	(0.7–7.8)	0.15
Autoimmune	NA^a^			NA^a^		
Fatty liver	1.6	(0.5–5.5)	0.4	3.3	(0.8–14.3)	0.11
Cryptogenic	2.1	(0.2–18.8)	0.5	2.6	(0.2–31)	0.5
Hepatocellular carcinoma	1.4	(0.7–2.9)	0.4	2	(0.8–5)	0.11

*Abbreviations*: *CCI* Charlson Comorbidity Index (excluding liver disease and liver cancer), *MELD-Na* Model for End-stage Liver Disease with sodium modification, *NA* not applicable

Footnotes: ^a^ no patients in the analysis or with the endpoint to analyze

On closer examination, the impact of CCI on 90-day mortality was largely related to increasing risk in patients with MELD-Na above the median value of 17. In patients with MELD-Na ≥ 18, 90-day mortality was 12% with CCI = 0, 22% with CCI = 1–2 and 33% with CCI > 2, (*p* = 0.03)). Whereas in patients with MELD-Na ≤ 17, 90-day mortality was 1% with CCI = 0, 1% with CCI = 1–2 and 4% with CCI > 2, (*p* = 0.5)). Patients with MELD-Na ≥ 18 also had increasing 90-day mortality with higher categories of CIRCOM (11% with CIRCOM = 0, 18% with CIRCOM = 1–2 and 39% with CIRCM> 2 (highest decile for CIRCOM), (*p* = 0.002)).

### The impact of comorbidity burden on overall survival

The median overall follow-up to time of death, LT or last follow-up was 332 days (IQR 161, 919). During this time 186 patient died with a median time to death of 303 days (IQR 126, 822). The median follow-up in 54 patients alive at last contact without LT was 6 years (IQR 0.6, 6.5), and in 100 patients who underwent LT was 5.7 years (IQR 4, 7.3). Post-LT patient and graft survival rates were both 90% at 1 year and 81% at 5 years. Patients who died without LT had higher comorbidity burden than those who survived or underwent LT (Table 3). Compared to surviving patients, they were also more commonly male with viral liver disease, higher MELD-Na and HCC.

**Table 3:** Baseline demographic and clinical characteristics of the patients evaluated for liver transplantation (LT) for complications of cirrhosis among patients who; (i) were alive at last follow up without LT, (ii) died without LT or (iii) underwent LT. Data are shown as mean ± standard deviation or percentage

Demographic and clinical characteristics	Survived without LT ***n*** = 54	Died without LT ***n*** = 186	Underwent LT ***n*** = 100^**a**^	***P***-value
Age	54 ± 10	57 ± 9	56 ± 10	0.14
Gender (male) (%)	43	64	71	0.002
Race (%)				
White	87	91	91	0.4
Black	4	4	6	
Hispanic	6	4	1	
Asian	None	1	1	
Other	3	None	1	
Body mass index	28 ± 6	30 ± 7	30 ± 6	0.4
Etiology of liver disease (%)				
Alcohol	29	34	30	0.001
Alcohol and viral	12	21	10	
Viral	29	18	13	
Autoimmune	9	2	17	
Fatty liver	19	20	24	
Cryptogenic	2	2	3	
MELD	13.9 ± 4.4	17.7 ± 7.1	18.1 ± 5.9	< 0.001
Hepatocellular carcinoma (%)	7	27	25	0.01
CCI (mean)	0.6 ± 0.9	1.1 ± 1.3	0.6 ± 0.8	< 0.001
(median (IQR))	0 (IQR 0,1)	1 (IQR 0,2)	0 (IQR 0,1)	
CCI Category (%)				
CCI = 0	59	34	56	0.001
CCI = 1–2	33	50	38	
CCI > 2	8	16	6	
CIRCOM	0.6 ± 0.8 0 (IQR 0,1)	1.1 ± 1.3 1 (IQR 0,2)	0.4 ± 0.9 0 (IQR 0,1)	< 0.001

*Abbreviations*: *CCI* cirrhosis-modified Charlson Comorbidity Index (excluding liver disease and liver cancer), *IQR* interquartile range, *MELD-Na* Model for End-stage Liver Disease with sodium modification

Footnotes: ^a^ includes one patient who underwent LT at another center

**Fig. 1:**
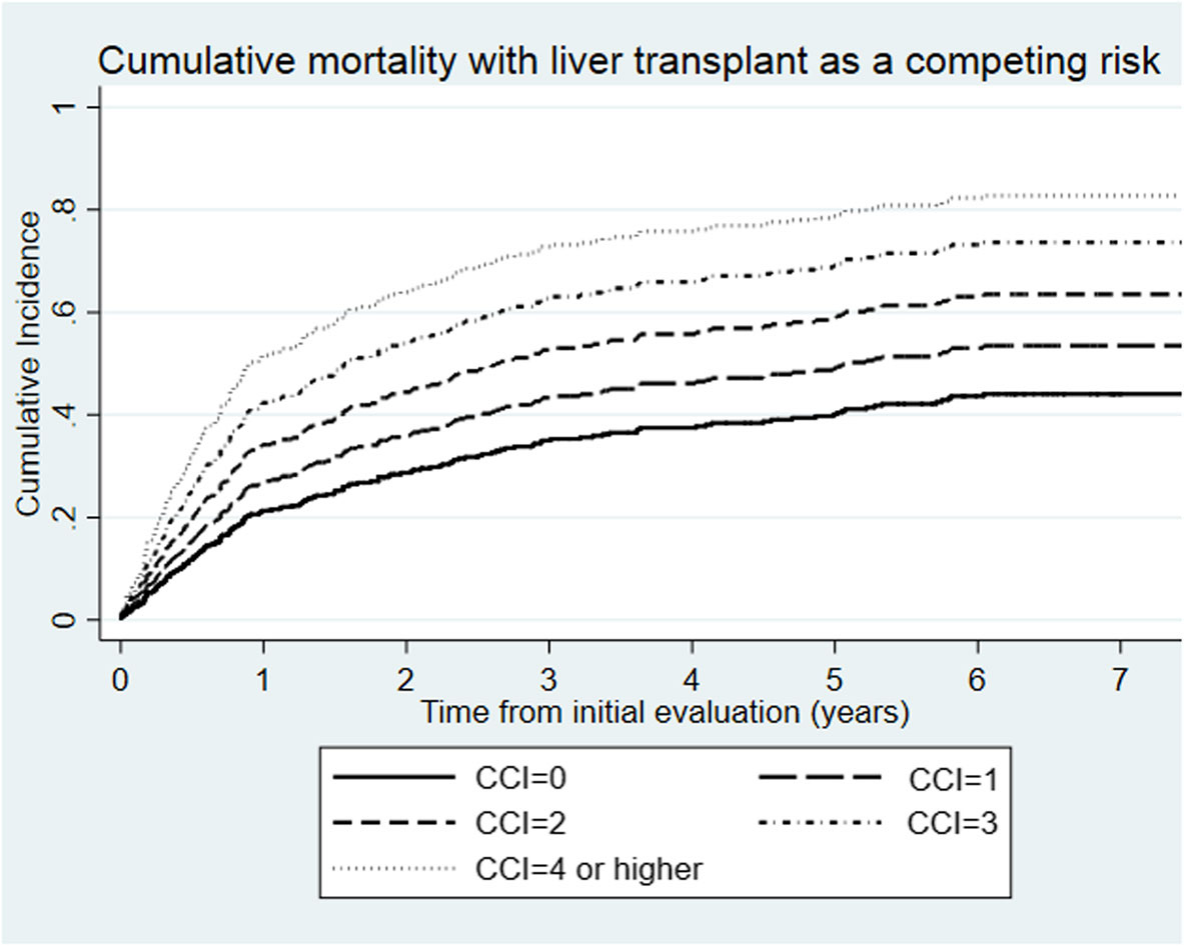
Overall mortality in the competing risk model (liver transplant as the competing risk) stratified by comorbidity burden using the Charlson Comorbidity Index (CCI) (excluding liver disease and liver cancer). The analysis was adjusted for age, gender, race, model for endstage liver disease with sodium modification, body mass index, liver disease etiology and presence of hepatocellular carcinoma. The sub-hazard ratio for each 1-point increment in CCI was 1.24 (95%confidence interval 1.1–1.4)

The CCI was associated with increased overall mortality on multivariable competing risk regression analysis (Table 4). The risk-adjusted cumulative incidence of mortality increased with each CCI point **(**Fig. 1**)**. Extra-hepatic comorbidity burden as measured by CIRCOM was also associated with overall mortality (adjusted Sub-HR 1.3, 95%CI 1.2–1.5). The CCI was associated with overall mortality irrespective of baseline MELD-Na (adjusted sub-HR 1.3 (95%CI 1.1–1.6) with MELD-Na ≤ 17, and adjusted sub-HR 1.2 (95%CI 1.1–1.4) with MELD-Na ≥ 18).

**Table 4:** The factors associated with overall mortality in the competing-risk regression analysis with liver transplantation as the competing risk

Factor	Univariable analysis			Multivariable analysis		
	Sub-Hazard ratio	95% confidence interval	***P***-value	Sub-Hazard ratio	95% confidence interval	***P***-value
CCI	1.25	(1.14–1.38)	< 0.001	1.24	(1.1–1.4)	< 0.001
MELD-Na	1.05	(1.02–1.08)	0.002	1.05	(1.02–1.09)	0.001
Age	1.01	(0.99–1.02)	0.2	1.01	(0.99–1.03)	0.2
Male gender	1.2	(0.9–1.6)	0.3	0.94	(0.7–1.3)	0.7
Body mass index	1.01	(0.98–1.03)	0.5	1.01	(0.98–1.03)	0.7
Race (reference white)						
Black	0.9	(0.5–1.7)	0.7	0.8	(0.4–1.7)	0.5
Hispanic	1.5	(0.7–3.3)	..3	1.4	(0.5–3.5)	0.6
Asian	1.3	(0.4–5)	0.7	3.1	(1.6–5.7)	0.001
Etiology liver disease (Reference alcohol)						
Alcohol and viral	1.7	(1.02–2.7)	0.03	1.6	(0.96–2.67)	0.07
Viral	1.2	(0.8–1.8)	0.4	1.1	(0.7–1.8)	0.6
Autoimmune	0.2	(0.08–0.6)	0.006	0.2	(0.09–0.7)	0.009
Fatty liver	1.1	(0.7–1.7)	0.7	0.9	(0.6–1.6)	0.8
Cryptogenic	1.1	(0.5–2.7)	0.8	0.7	(0.4–2.2)	0.8
Hepatocellular carcinoma	1.3	(0.9–1.7)	0.14	1.1	(0.7–1.6)	0.7

*Abbreviations*: *CCI* Charlson Comorbidity Index (excluding liver disease and liver cancer), *MELD-Na* Model for End-stage Liver Disease with sodium modification, *NA* not applicable

### Individual comorbidities versus comorbidity burden and mortality

We examined the association of individual components of the CCI and CIRCOM with 90-day and overall mortality using univariable models due to small numbers of patients with each condition (Table 5). Only renal disease defined by a creatinine≥1.5 mg/dL (included in CIRCOM and contributes to MELD-Na) was associated with 90-day mortality. The conditions common to CCI and CIRCOM that were associated with overall mortality included congestive heart failure, renal disease and metastatic malignancy. The conditions included in CCI, but not CIRCOM, that were associated with overall mortality included chronic obstructive pulmonary disease, diabetes mellitus with complications, cerebrovascular disease, connective tissue disease and acquired immune deficiency syndrome. At least one of these conditions was observed in 27.1% of patients. The condition included in CIRCOM, but not CCI, that was associated with overall mortality was substance abuse (excluding alcohol) observed in 19.6% of patients.

**Table 5:** The association of individual components of the Charlson Comorbidity Index (excluding liver disease and liver cancer) and CIRCOM with 90-day and overall mortality in the unadjusted analyses

Comorbid conditions	Analysis of 90-day mortality ***n*** = 332				Analysis of overall mortality ***n*** = 340			
	Patients with condition (%)	Hazard ratio	95% confidence interval	***P***-value	Patients with condition (%)	Sub-Hazard ratio	95% confidence interval	***P***-value
Myocardial infarction^b c^	1.8	NA^a^			1.8	1.3	(0.8–2.2)	0.3
Coronary artery disease (no infarction)	18.4	0.4	(0.1–1.4)	0.17	18.5	1.1	(0.8–1.5)	0.7
Congestive heart failure^b c^	4.2	0.8	(0.1–5.6)	0.8	4.4	2	(1.4–2.9)	< 0.001
Peripheral vascular disease^b c^	3.3	NA^a^			3.2	1.1	(0.6–2.2)	0.7
Cerebrovascular disease^b^	2.7	1.2	(0.16–8.8)	0.9	2.7	2	(1.1–3.4)	0.015
Dementia^b^	0.9	4 (	0.5–29)	0.17	1.2	2	(0.5–7.6)	0.3
Chronic obstructive pulmonary disease^b^	15.1	1.5	(0.6–3.4)	0.3	13.5	1.6	(1.1–2.2)	0.007
Connective tissue disease^b^	3.6	3.1	(0.9–10)	0.06	3.8	1.9	(1.1–3.4)	0.03
Peptic ulcer disease^b^	6	1.7	(0.5–5.5)	0.4	5.3	1.5	(0.8–2.6)	0.2
Diabetes without complications^b^	23.8	0.6	(0.2–1.4)	0.2	24.4	0.95	(0.7–1.3)	0.8
Diabetes with complications^b^	8.1	0.7	(0.2–3)	0.2	7.9	1.6	(1.1–2.4)	0.016
Hemiplegia^b^	0.6	NA^a^			0.6	0.9	(0.1–6.1)	0.9
Renal disease (creatinine > 3 mg/dL)^b^	2.1	1.5	(0.2–11)	0.7	1.8	5.1	(0.9–30)	0.07
Renal disease (creatinine ≥1.5 mg/dL)^c^	15.4	4.2	(2.1–8.5)	< 0.001	15.9	2.4	(1.6–3.5)	< 0.001
Leukemia^b c^	None	NA			None	NA		
Lymphoma^b c^	None	NA			None	NA		
Non-metastatic solid tumor^b c^	2.3	2.4	(0.6–10.1)	0.2	2.1	1.8	(06–5)	0.3
Metastatic solid tumor^b c^	0.3	NA^a^			0.3	6.8	(5.1–9.1)	< 0.001
AIDS^b^	0.3	NA^a^			0.3	4.1	(3.3–5.2)	< 0.001
Epilepsy^c^	3.3	NA^a^			3.5	1.1	(0.6–2.3)	0.7
Substance abuse other than alcoholism^v^	19.9	1.3	(0.6–2.9)	0.5	19.4	1.5	(1.1–2)	0.018

*Abbreviations*: *AIDS* acquired immune deficiency syndrome, *CCI* Charlson Comorbidity Index (excluding liver disease and liver cancer); HR, *CI* confidence interval; hazard ratio, *LT* liver transplant, *NA* not applicable

^a^No patients in the analysis met this endpoint

^b^Scored in the CCI

^c^Scored in the CIRCOM

### The impact of comorbidity burden on liver transplant eligibility

After the initial visit, 40 patients were deemed too early for LT and 38 patients chose not to pursue LT, and did not complete testing. Half of these patients were female, with mean MELD < 15, mean CCI < 1, and < 10% had HCC (Supplemental Table 2). Additionally, 48 patients died before completing transplant evaluation and, 28 did not complete evaluation. These patients were more frequently male, with mean MELD> 15 and mean CCI > 1 (Supplemental Table 2). The most common barriers for LT eligibility in patients attempting but not completing LT evaluation were advanced HCC, morbid obesity and active substance abuse which are not reflected in CCI, although substance abuse is included in CIRCOM (Supplemental Table 3).

One hundred and seventy-seven patients completed the evaluation process and were discussed formally in the transplant selection committee. They were predominantly male (69%), with mean baseline MELD 17 ± 5.8, mean CCI 0.98 ± 1.26, and 31% had HCC (Supplemental Table 2). Of these 177 comprehensively evaluated patients 120 were approved for listing by the transplant selection committee and 57 were deemed ineligible. The CCI was inversely associated with LT eligibility in the risk-adjusted model, whereas male gender and autoimmune liver disease were associated with LT eligibility (Table 6). Factors not associated with LT eligibility included age, MELD, BMI, race, and HCC. Extra-hepatic comorbidity burden was also inversely associated with LT-eligibility in the risk-adjusted analysis when assessed using CIRCOM (adjusted HR 0.74, 95%CI 0.6–0.91, *p* = 0.004).

**Table 6:** The factors associated with liver transplant (LT) eligibility by Cox regression analysis in 177 patients completing work-up for LT

Factor	Univariable analysis			Multivariable analysis		
	Hazard Ratio	95% confidence interval	***P***-value	Hazard Ratio	95% confidence interval	***P***-value
CCI	0.64	(0.53–0.78)	< 0.001	0.63	(0.51–0.79)	< 0.001
MELD committee	0.99	(0.96–1.01)	0.2	1	(0.97–1.03)	0.96
Age	1.002	(0.98–1.025)	0.9	1	(0.97–1.03)	0.9
Male gender	1.3	(0.9–1.9)	0.16	2.3	(1.5–3.7)	< 0.001
Body mass index	0.98	(0.96–1.01)	0.3	0.99	(0.96–1.03)	0.7
Race (reference white)						
Black	0.71	(0.3–1.2)	0.4	1.1	(0.4–2.6)	0.9
Hispanic	1.2	(0.4–3.8)	0.8	1.8	(0.5–6.2)	0.4
Asian	4.6	(1.1–18.8)	0.036	2	(0.5–10.6)	0.4
Etiology of liver disease (Reference alcohol)						
Alcohol and viral	0.7	(0.3–1.3)	0.2	0.6	(0.3–1.2)	0.16
Viral	1.1	(0.6–1.9)	0.8	1.1	(0.6–2.1)	0.7
Autoimmune	2.1	(1.1–4)	0.03	2.4	(1.2–5.2)	0.02
Fatty liver	1.1	(0.6–2)	0.9	1.6	(0.8–3.2)	0.17
Cryptogenic	1.2	(0.4–3.7)	0.7	0.9	(0.3–3.1)	0.9
Hepatocellular carcinoma	0.65	(0.43–0.98)	0.038	1	(0.6–1.7)	0.98

*Abbreviations*: *CCI* Charlson Comorbidity Index (excluding liver disease and liver cancer), *MELD* Model for End-stage Liver Disease with sodium modification, *NA* not applicable

The most common barriers to LT eligibility in the 57 patients were cardiac, advanced HCC, psychosocial concerns and debilitation (Supplemental Table 3). Cardiac factors prohibitive of LT in 16 patients were related to coronary artery disease in 13 and uncontrolled arrhythmias in 2, neither of which is reflected in CCI or CIRCOM. The individual comorbid conditions that impacted LT eligibility in the unadjusted analysis included coronary artery disease without infarction, congestive heart failure, peptic ulcer disease, diabetes with complication, renal disease and substance abuse (Supplemental Table 4). Only 120 patients were waitlisted of whom 100 underwent LT. Both CCI and CIRCOM did not impact waitlist or post-LT survival in the risk adjusted analyses, although the number of patients analyzed was small (data not shown).

## Discussion

The main novel finding in this study was that extra-hepatic comorbidity burden adversely impacted 90-day mortality among patients with cirrhosis evaluated for LT. This interplay between CCI and MELD-Na for short-term mortality was largely attributed to increased risk in patients with MELD-Na ≥ 18. Interestingly, the inflection threshold for improving survival benefit for LT in cirrhosis was recently demonstrated at a MELD-Na range of 18–20 [13]. In other words, extra-hepatic comorbidity burden appears to amplify short-term mortality in patients with cirrhosis who benefit the most from LT, and may be an important consideration for LT urgency in these patients.

The association of CCI and 90-day mortality persisted even when excluding patients with moderate to severe renal disease that may confound the association due to contribution of creatinine to MELD-Na. Beyond renal dysfunction, which is already reflected in higher MELD-Na, no component conditions of CCI or CIRCOM were associated with 90-day mortality. Therefore, the overall burden of extrahepatic comorbidities, as measured by CCI or CIRCOM, rather than specific conditions were the drivers of risk for 90-day mortality. This underscores the potential utility of an aggregate comorbidity burden score, beyond individual conditions, in risk assessment for patients with advanced liver disease.

Liver transplantation was an important factor in the consideration of overall mortality, with expected high rates of patient survival at 5 years post LT. This necessitated the assessment of overall mortality with competing-risk regression as described by Fine and Gray [14]. Since few patients underwent LT within 90-days of initial evaluation the short-term mortality risk with CCI could not be attributed to the impact of CCI on transplant eligibility. However, comorbidities may represent barriers for LT candidacy, and not surprisingly comorbidity burden was higher in patient who died compared with those undergoing LT or surviving without LT. This data suggests that comorbidity may carry dual risks for patients with cirrhosis. An increased short-term risk in those with more advanced disease, and longer-term risk of both mortality and potential barriers to life-saving LT as their liver disease detriorates11–12%.

The examination of the impact of individual comorbid conditions also demonstrated that no scoring system captured all conditions that appeared to impact overall mortality in the cohort. The CCI does not measure substance abuse which is included in CIRCOM, and CIRCOM does not measure 5 conditions included in CCI (chronic obstructive pulmonary disease, diabetes mellitus with complications, cerebrovascular disease, connective tissue disease and acquired immune deficiency syndrome). These differences would have impacted comorbidity burden measurement in 19.4 and 27.1% of patients, respectively. Both scores still performed well as measures of extra-hepatic comorbidity burden with very similar adjusted hazard and sub-hazard ratios. It is not within the scope nor the intent of this study to suggest modifications of comorbidity scoring systems, but we acknowledge the interesting differences between these scores that could impact 1 in 4 to 5 patients with advanced cirrhosis.

The CCI was developed as a continuous scale or variable and the categorical descriptive analysis warrants discussion. A CCI = 0 reflects a physiologically important reference group with no or very low comorbidity burden. A CCI > 2 highlighted the risks associated with the highest decile of extra-hepatic comorbidity burden, e.g. in patients with higher MELD-Na. Notably, other studies have demonstrated increased mortality in patients with cardiac disease and in patients without cirrhosis presenting with acute liver injury, using the same threshold [9, 15, 16]. However, the ideal thresholds if any for estimating CCI related risk in the context of pre-LT outcomes may be refined with additional studies.

A higher comorbidity burden was observed in patients with fatty and cryptogenic liver disease. The latter is commonly attributed to undiagnosed fatty liver disease, which is known to be associated with extra-hepatic comorbidities and increasingly driving the need for LT in the United Sates [17, 18]. While we observed age differences according to disease etiology, extra-hepatic comorbidity burden did not correlate with age per se, suggesting that the observed associations were mainly related to differences in disease associations rather than older age alone. Gender-based disparities in LT eligibility have been described with increased pre-LT and waitlist mortality in women [19, 20]. Additionally, gender-based differences in medical comorbidities have been described in hospitalized patients with higher rates of diabetes and connective tissue disease but without evident differences in hospital mortality [21]. In our cohort, there were no gender-based differences in CCI, although women had a trend for more frequent connective tissue disease. Higher 90-day mortality was observed in men, but that association was fully attenuated in the risk-adjusted analysis.

In practice, LT candidate selection is impacted by individual clinically relevant comorbidities and psychosocial considerations rather than a comorbidity burden score such as CCI. There is a dearth of data in this area, but Arya et al. have demonstrated that less than half of patients evaluated for LT were deemed ineligible, mainly for being too early, medical conditions or addiction problems [3]. We observed similar outcomes in our cohort and explored the role of comorbid conditions in detail as potential barriers to LT. Extra-hepatic comorbidity burden (CCI) was associated with LT-ineligibility and multiple comorbid conditions were individually associated with LT-ineligibility. However, the majority of barriers to LT in patients reviewed by the selection committee or in those not completing evaluation (coronary artery disease, obesity, being too ill or frail, advanced HCC or psychosocial issues such substance abuse) were not reflected in the CCI. Only 5 patients were LT-ineligible for other comorbidities (lung disease 2, and non-liver malignancy 3) which were reflected in CCI. The CCI components of diabetes with end organ damage and peptic ulcer disease were also associated with LT-ineligibility, but were not in of themselves contraindications for LT. In other words, conditions contraindicating LT were reflected in CCI in less than 10% of LT-ineligible patients. Therefore, measurement of extra-hepatic comorbidity burden was not a substitute for, but possibly complementary of, reasoned clinical judgment in determining LT candidacy.

The strengths of this study include the comprehensive characterization of comorbid conditions and the long-term follow up of patients with cirrhosis consecutively evaluated for LT. The assessment of extra-hepatic comorbidity burden using two validated systems (CCI and CIRCOM) and convergence of the observed associations also support the clinical premise of the study. The limitations of the study include the retrospective design, limited racial diversity, the absence of comprehensive information on socioeconomic status, and in some cases limited follow-up intervals in patients not undergoing LT. Finally, the use of CCI and CIRCOM in this study was demonstrative of the impact of comorbidity burden on mortality and was not a judgement of superiority of a specific score over other available instruments.

## Conclusion

In summary, this study demonstrates and quantifies the risk associated with extra-hepatic comorbidity burden with increased short and long-term mortality in patients with advanced cirrhosis. These data are timely, given an ageing population and increasing burden of fatty liver disease and comorbid conditions on transplant and other healthcare resources. Assessment of comorbidity burden may identify a subset of patients with the highest mortality risk and increased LT urgency. If validated, standardized measurement of extra-hepatic comorbidity burden may also be an important modifier of mortality risk models and mortality-related healthcare metrics in cirrhosis. Further studies are needed to confirm these findings, and to determine the ideal comorbidity scoring system and thresholds in patients with advanced cirrhosis.

## Supplementary information

**Additional file 1: Supplemental Table 1.** The extra-hepatic conditions and their relative weights comprising the Charlson Comorbidity Index (excluding liver disease and hepatocellular carcinoma) and the cirrhosis comorbidity score (CIRCOM). **Supplemental Table 2.** Baseline demographic and clinical characteristics of the patients evaluated for liver transplantation (LT) for complications of cirrhosis among patients who; (i) were deemed too early for LT (ii) did not want to pursue LT (iii) did not complete LT evaluation (iv) died before competing LT evaluation, or (v) completed LT evaluation with selection committee review. Data are shown as mean ± standard deviation or percentage. **Supplemental Table 3.** The barriers to liver transplant (LT) eligibility in 28 patients not completing LT evaluation and 57 patients completing evaluation but not approved for LT after selection committee review. Data shown as numbers (and percentages for the main categories of barriers). **Supplemental Table 4.** The univariable Cox regression analyses of association of individual components of the Charlson Comorbidity Index (excluding liver disease and liver cancer) and CIRCOM, with the study endpoints of liver transplant eligibility and post liver transplant survival.

## Data Availability

Per the Indiana University institutional review board, data is to be deidentified and stored on a secure server, but no permission to store on a publicly accessible server. Specific data points requests, excluding any patient identifiers, directed to the corresponding author will be considered and honored.

## Abbreviations

BMI: Body mass index
CCI: Charlson Comorbidity Index
CIRCOM: Cirrhosis Comorbidity score
CI: Confidence interval
HCC: Hepatocellular carcinoma
HR: Hazard ratio
LT: Liver transplantation
MELD: Model for endstage liver disease
MELD-Na: Model for endstage liver disease with sodium modification

## References

[1] Bianchi G, Marchesini G, Zoli M (1994). Prognostic significance of diabetes in patients with cirrhosis. Hepatology.

[2] Jepsen P, Vilstrup H, Lash TL. Development and validation of a comorbidity scoring system for patients with cirrhosis. Gastroenterology, 2014. 146:147–56 quiz e15–6. 10.1053/j.gastro.2013.09.019 24055278

[3] Arya A, Hernandez-Alejandro R, Marotta P (2013). Recipient ineligibility after liver transplantation assessment: a single Centre experience. Can J Surg.

[4] Volk ML, Hernandez JC, Lok AS (2007). Modified Charlson comorbidity index for predicting survival after liver transplantation. Liver Transpl.

[5] Charlson ME, Pompei P, Ales KL (1987). A new method of classifying prognostic comorbidity in longitudinal studies: development and validation. J Chronic Dis.

[6] Lee DS, Austin PC, Rouleau JL (2003). Predicting mortality among patients hospitalized for heart failure: derivation and validation of a clinical model. JAMA.

[7] Sanchis J, Nunez J, Bodi V (2011). Influence of comorbid conditions on one-year outcomes in non-ST-segment elevation acute coronary syndrome. Mayo Clin Proc.

[8] Sanchis J, Soler M, Nunez J (2019). Comorbidity assessment for mortality risk stratification in elderly patients with acute coronary syndrome. Eur J Intern Med.

[9] Ghabril M, Gu J, Yoder L, et al. Development and validation of model consisting of comorbidity burden to calculate risk of death within 6 months for patients with suspected drug-induced liver injury. Gastroenterology. 2019;157(5):1245–52.e3. 10.1053/j.gastro.2019.07.006 PMC6815697 31302142

[10] O'Brien BC, Harris IB, Beckman TJ (2014). Standards for reporting qualitative research: a synthesis of recommendations. Acad Med.

[11] Kim WR, Biggins SW, Kremers WK (2008). Hyponatremia and mortality among patients on the liver-transplant waiting list. N Engl J Med.

[12] Xu Y, Li N, Lu M (2017). Comparison of risk adjustment methods in patients with liver disease using electronic medical record data. BMC Gastroenterol.

[13] Nagai S, Chau LC, Schilke RE (2018). Effects of allocating livers for transplantation based on model for end-stage liver disease-sodium scores on patient outcomes. Gastroenterology.

[14] Fine JP, Gray RJ (1999). A proportional hazards model for the subdistribution of a competing risk. J Am Stat Assoc.

[15] Formiga F, Moreno-Gonzalez R, Chivite D (2018). High comorbidity, measured by the Charlson comorbidity index, associates with higher 1-year mortality risks in elderly patients experiencing a first acute heart failure hospitalization. Aging Clin Exp Res.

[16] Fraccaro P, Kontopantelis E, Sperrin M (2016). Predicting mortality from change-over-time in the Charlson comorbidity index: a retrospective cohort study in a data-intensive UK health system. Medicine (Baltimore).

[17] Pham T, Dick TB, Charlton MR (2016). Nonalcoholic fatty liver disease and liver transplantation. Clin Liver Dis.

[18] Wong RJ, Aguilar M, Cheung R (2015). Nonalcoholic steatohepatitis is the second leading etiology of liver disease among adults awaiting liver transplantation in the United States. Gastroenterology.

[19] Moylan CA, Brady CW, Johnson JL (2008). Disparities in liver transplantation before and after introduction of the MELD score. JAMA.

[20] Cullaro G, Sarkar M, Lai JC (2018). Sex-based disparities in delisting for being "too sick" for liver transplantation. Am J Transplant.

[21] Rubin JB, Srisengfa YT, Albhaisi S, et al. Clin Gastroenterol Hepatol. 2019;S1542–3565(19):31094–8.

