# Supplementary material for: Extra-hepatic comorbidity burden significantly increases 90-day mortality in patients with cirrhosis and high model for endstage liver disease
Source: BMC Gastroenterol. 2020 Sep 16;20:302. doi: 10.1186/s12876-020-01448-z (PMC7493147; doi:10.1186/s12876-020-01448-z)
Supplement: Supplementary file 1 — Additional file 1: Supplemental Table 1. The extra-hepatic conditions and their relative weights comprising the Charlson Comorbidity Index (excluding liver disease and hepatocellular carcinoma) and the cirrhosis comorbidity score (CIRCOM). Supplemental Table 2. Baseline demographic and clinical characteristics of the patients evaluated for liver transplantation (LT) for complications of cirrhosis among patients who; (i) were deemed too early for LT (ii) did not want to pursue LT (iii) did not complete LT evaluation (iv) died before competing LT evaluation, or (v) completed LT evaluation with selection committee review. Data are shown as mean ± standard deviation or percentage. Supplemental Table 3. The barriers to liver transplant (LT) eligibility in 28 patients not completing LT evaluation and 57 patients completing evaluation but not approved for LT after selection committee review. Data shown as numbers (and percentages for the main categories of barriers). Supplemental Table 4. The univariable Cox regression analyses of association of individual components of the Charlson Comorbidity Index (excluding liver disease and liver cancer) and CIRCOM, with the study endpoints of liver transplant eligibility and post liver transplant survival. [file 12876_2020_1448_MOESM1_ESM.docx]

**SUPPLEMENTAL DATA**

| **Extra-hepatic comorbid conditions (score weight)** | **Charlson Comorbidity index**  (Points added if present) | **CIRCOM**  (Possible points based on algorithm and presence of other conditions) |
| --- | --- | --- |
| Acquired Immune Deficiency Syndrome | 6 points | Not included |
| Metastatic solid tumor | 6 points | 5 points |
| Non-metastatic solid tumor (Exclude non-melanoma malignant neoplasm of skin) | 2 points | 0,1 or 3 points |
| Lymphoma | 2 points | 0, 1 or 3 points |
| Leukemia | 2 points | 0, 1 or 3 points |
| Moderate or severe renal disease | 2 points  Serum creatinine of > 3 mg/dl or dialysis | 0, 1 or 3 points |
| Hemiplegia or paraplegia | 2 points | Not included |
| Diabetes with end organ damage | 2 points | Not included |
| Diabetes without complications | 1 point | Not included |
| Any diabetes | Scored by presence of end organ damage, see above | Not included |
| Peptic ulcer disease | 1 point | Not included |
| Connective tissue disease | 1 point | Not included |
| Chronic obstructive pulmonary disease | 1 point | Not included |
| Dementia | 1 point | Not included |
| Cerebrovascular disease or transient ischemic attack (TIA) | 1 point | Not included |
| Peripheral Vascular disease | 1 point | 0 or 1 point |
| Congestive heart failure | 1 point | 0 or 1 point |
| Myocardial infarction | 1 point  Excludes coronary artery disease without myocardial infarction | 0, 1 or 3 points |
| Epilepsy | Not included | 0 or 1 point |
| Substance abuse other than alcoholism | Not included | 0 or 1 point |

**Supplemental table 1.** The extra-hepatic conditions and their relative weights comprising the Charlson Comorbidity Index (excluding liver disease and hepatocellular carcinoma) and the cirrhosis comorbidity score (CIRCOM).

.

**Supplemental Table 2.** Baseline demographic and clinical characteristics of the patients evaluated for liver transplantation (LT) for complications of cirrhosis among patients who; (i) were deemed too early for LT (ii) did not want to pursue LT (iii) did not complete LT evaluation (iv) died before competing LT evaluation, or (v) completed LT evaluation with selection committee review. Data are shown as mean ± standard deviation or percentage.

| **Demographic and clinical characteristics** | **Patients deemed too early for LT**  **n=40** | **Did not want to pursue LT**  **n=38** | **Did not complete LT evaluation**  **n=28** | **Died before completing LT evaluation**  **n=48** | **completed LT evaluation and committee review**  **n=177** |
| --- | --- | --- | --- | --- | --- |
| Age | 56±10 | 57±10 | 56±9 | 55±10 | 57±9 |
| Gender (male) (%) | 45 | 53 | 75 | 63 | 69 |
| Race (%)  White  Black  Hispanic  Asian  Other | 87  3  7  None  3 | 89  8  None  3  None | 86  7  7  None  None | 92  None  8  None  None | 91  5  2  1  1 |
| Body mass index | 30±6 | 31±7 | 31±8 | 29±8 | 30±6 |
| Etiology of liver disease (%)  Alcohol  Alcohol and viral  Viral  Autoimmune  Fatty liver  Cryptogenic | 24  8  37  8  21  3 | 21  13  40  5  16  5 | 29  25  21  21  4  None | 21  23  31  None  23  2 | 14  16  31  12  22  3 |
| MELD | 11.7±4 | 14.6±5.5 | 16.5±7 | 22.7±7 | 17±5.8 |
| Hepatocellular carcinoma (%) | 5 | 9 | 29 | 10 | 31 |
| CCI | 0.8±1 | 0.9±1 | 1.43±0.1.57 | 1.2±1.3 | 0.98±1.26 |
| CCI Category (%)  CCI = 0 | 52 | 39 | 25 | 37 | 48 |
| CCI = 1-2 | 38 | 50 | 57 | 48 | 41 |
| CCI > 2 | 10 | 11 | 18 | 15 | 11 |
| CIRCOM | 0.75±1.1 | 0.82±1.1 | 0.89±1.1 | 1.3±1.4 | 0.7±1.1 |

Abbreviations: CCI, Charlson Comorbidity Index (excluding liver disease and liver cancer); LT, liver transplantation; MELD, Model for End-stage Liver Disease with sodium modification.

**Supplemental Table 3.** The barriers to liver transplant (LT) eligibility in 28 patients not completing LT evaluation and 57 patients completing evaluation but not approved for LT after selection committee review. Data shown as numbers (and percentages for the main categories of barriers).

| **Barriers** | **Did not complete LT evaluation**  **n=28** | **Not LT eligible after evaluation and committee review**  **n=57** |
| --- | --- | --- |
| **Clinical barriers** | **17 (61%)** | **42 (74%)** |
| Cardiac | 1 | 15 |
| Pulmonary | None | 2 |
| Advanced HCC | 6 | 10 |
| Non-liver malignancy | 2 | 3 |
| Too sick or frail | 2 | 9 |
| Morbid obesity | 6 | 3 |
| **Psychosocial barriers** | **8 (29%)** | **13 (23%)** |
| Active substance use | 5 | 6 |
| Did not complete substance abuse program | 1 | 5 |
| Lack of social support or compliance | 2 | 2 |
| **Other barriers** | **3 (11%)** | **2 (3%)** |
| Loss of insurance | 2 | 2 |
| Pursued evaluation elsewhere | 1 | None |

Abbreviations: HCC, hepatocellular carcinoma; LT, liver transplantation.

**Supplemental Table 4.** The univariable Cox regression analyses of association of individual components of the Charlson Comorbidity Index (excluding liver disease and liver cancer) and CIRCOM, with the study endpoints of liver transplant eligibility and post liver transplant survival.

|  | **Analysis of LT eligibility**  **n=177** | | |
| --- | --- | --- | --- |
| **Comorbid conditions** | Patients with condition (%) | **HR (95%CI)** | **P value** |
| Myocardial infarction † ¥ | 1.7 | 0.5 (0.1 – 3.7) | 0.5 |
| Coronary artery disease (no infarction) | 25.4 | 0.5 (0.3 – 0.8) | 0.002 |
| Congestive heart failure † ¥ | 3.3 | 0.14 (0.02 – 0.97) | 0.047 |
| Peripheral vascular disease † ¥ | 3.3 | 0.4 (0.1 – 1.4) | 0.15 |
| Cerebrovascular disease† | 2.3 | 0.2 (0.03 – 1.7) | 0.16 |
| Dementia † | None | NA* |  |
| Chronic obstructive pulmonary disease † | 12.4 | 0.6 (0.3 – 1.05) | 0.07 |
| Connective tissue disease † | 2.3 | 0.9 (0.3 – 2.8) | 0.9 |
| Peptic ulcer disease † | 6.8 | 0.35 (0.2 – 0.9) | 0.02 |
| Diabetes without complications † | 18.6 | 1.1 (0.7 – 1.7) | 0.7 |
| Diabetes with complications † | 10.1 | 0.24 (0.1 – 0.6) | 0.002 |
| Hemiplegia † | 0.6 | 0.6 (0.1 – 4.3) | 0.6 |
| Renal disease (creatinine >3 mg/dL) † | 3.3 | 0.6 (0.2 – 1.6) | 0.3 |
| Renal disease (creatinine ≥ 1.5 mg/dL) ¥ | 16.3 | 0.5 (0.3 – 0.9) | 0.03 |
| Leukemia † ¥ | None | NA* |  |
| Lymphoma † ¥ | None | NA* |  |
| Non-metastatic solid tumor † ¥ | 3.3 | 1.1 (0.4 – 3) | 0.8 |
| Metastatic solid tumor † ¥ | 0.3 | NA* |  |
| AIDS † | 0.6 | NA* |  |
| Epilepsy ¥ | 1.7 | 1.4 (0.4 – 5.8) | 0.6 |
| Substance abuse other than alcoholism ¥ | 15.2 | 0.3 (0.2 – 0.6) | 0.001 |

Abbreviations: AIDS, acquired immune deficiency syndrome; CCI, Charlson Comorbidity Index (excluding liver disease and liver cancer); HR, CI, confidence interval; hazard ratio; LT, liver transplant; NA, not applicable.

Footnotes: * No patients in the analysis or with the endpoint to analyze

† Scored in the CCI

¥ Scored in the CIRCOM
